# Comparative Study of Electroless Copper Film on Different Self-Assembled Monolayers Modified ABS Substrate

**DOI:** 10.3390/ijms15046412

**Published:** 2014-04-15

**Authors:** Jiushuai Xu, Ruibin Fan, Jiaolong Wang, Mengke Jia, Xuanrui Xiong, Fang Wang

**Affiliations:** 1 College of Science, Northwest A&F University, Xinong Road No. 22, Yangling 712100, Shaanxi, China; E-Mails: xujiushuai@126.com (J.X.); xiafeng1202@126.com (R.F.); wjlong@nwsuaf.edu.cn (J.W.); jiamengke89@163.com (M.J.); 2 Graduate School of Environmental Earth Science, Hokkaido University, N10W5, Kita-ku, Sapporo, Hokkaido 060-0810, Japan; E-Mail: xiongxuanrui@ees.hokudai.ac.jp

**Keywords:** ABS resin, SAMs, electroless copper film, heterocyclic silane

## Abstract

Copper films were grown on (3-Mercaptopropyl)trimethoxysilane (MPTMS), (3-Aminopropyl)triethoxysilane (APTES) and 6-(3-(triethoxysilyl)propylamino)-1,3,5- triazine-2,4-dithiol monosodium (TES) self-assembled monolayers (SAMs) modified acrylonitrile-butadiene-styrene (ABS) substrate via electroless copper plating. The copper films were examined using scanning electron microscopy (SEM) and X-ray diffraction (XRD). Their individual deposition rate and contact angle were also investigated to compare the properties of SAMs and electroless copper films. The results indicated that the formation of copper nuclei on the TES-SAMs modified ABS substrate was faster than those on the MPTMS-SAMs and APTES-SAMs modified ABS substrate. SEM images revealed that the copper film on TES-SAM modified ABS substrate was smooth and uniform, and the density of copper nuclei was much higher. Compared with that of TES-SAMs modified resin, the coverage of copper nuclei on MPTMS and APTES modified ABS substrate was very limited and the copper particle size was too big. The adhesion property test demonstrated that all the SAMs enhanced the interfacial interaction between copper plating and ABS substrate. XRD analysis showed that the copper film deposited on SAM-modified ABS substrate had a structure with Cu(111) preferred orientation, and the copper film deposited on TES-SAMs modified ABS substrate is better than that deposited on MPTMS-SAMs or APTES-SAMs modified ABS resins in electromigrtion resistance.

## 1. Introduction

Acrylonitrile-butadiene-styrene (ABS) resin is an important engineering material for its excellent mechanical strength, high thermal stability, superduper impact resistance, glorious resistance to chemical reagents and some other properties [1]. Metalized ABS resin with outstanding properties of engineering plastic and metal has been widely used in cosmetic packaging, furniture decoration, and also possesses immense potential in automobile industry, electronic industry, petrolic industry and national defense field [2].

The conventional electroless copper plating method was directly to deposit copper layer on the ABS substrate which was pretreated to roughen the surface by plasma energy in order to form a mechanical interlock at the interface [3]. However, due to its inherently chemical hydrophobic, like some other polymers, ABS resin has poor adhesion property to metals. Some technologies for improving the coarseness of ABS resin surface have been adopted to enhance the adhesion between the ABS substrate and metal, such as ion bombardment, plasma treatment, acid treatments and corona discharge treatment [4–7]. Garcia *et al.* [8] used covalent grafting poly (acrylic Acid) on the surface of ABS substrate to improve adhesion strength between electroless copper and the resin, but the improvement was insufficient. Therefore, to obtain the electroless copper layer with huge cohesive force and uniformity is still a great challenge.

Our previous studies indicated that the electroless copper film fabricated under optimal technical conditions on self-assembled monolayers modified ABS resin surface was bright, non-porous, uniform, and had a better adhesion with ABS substrate [7,9]. Organic siloxane SAMs growing uniformly on resin substrate surface can serve as interfacial adhesion enhancer and modify surface properties. Through introducing functional groups to the terminal of monolayer, the chemical modification for the SAMs can be achieved, and the wetting [10], corrosion [11], etching [12] and some other characteristics can be controlled.

In this study, the ABS resin surface was firstly pretreated by corona discharge which was a electrical discharge at the surface of a conductor or between two conductors of the same transmission line, accompanied by ionization of the surrounding atmosphere, and then (3-Mercaptopropyl)trimethoxysilane (MPTMS), (3-Aminopropyl)triethoxysilane (APTES) and 6-(3-(triethoxysilyl)propylamino)-1,3,5-triazine- 2,4-dithiol monosodium (TES) self-assembled monolayers were fabricated on the pretreated ABS surface, respectively. The morphologies and structures of their electroless copper plating are characterized by scanning electron microscopy (SEM) and X-ray diffraction (XRD). Furthermore, their individual deposition rate and contact angle are also investigated to compare the properties of different siloxane SAMs and electroless copper layers.

## 2. Results and Discussion

### 2.1. Deposition Rate and Contact Angle

The deposition rate of copper on different SAMs covered ABS resin is shown in Table 1. It can be seen that the formation of copper atom nucleation on the substrate modified by TES-SAM is easier and faster, and the copper plating is the thickest. Compared with that on TES-SAMs modified substrate, the copper deposition rate on MPTMS-SAM modified surface decreased, and the deposition on APTES-SAM modified substrate was the slowest. It could be supposed that the sulfhydryl terminal group was more beneficial to coordinate with copper atom than amino terminal group.

The contact angle depends both on the surface morphology and chemical structure. The change of these factors would modify its surface energy which led to the change in the contact angle. Therefore, the surfaces of ABS substrate modified by different SAMs had different contact angle due to their difference in the surface energy. The related research indicated that the initial copper atoms could easily aggregate on the surface of low surface energy, and the average size of grains would be bigger [13]. However, the nucleation took place following, the so-called island or Volmer-Weber growth mode [14], where small clusters were nucleated directly onto the surface and then grew into islands of the condensed phase. Moreover, the previous investigation suggested that the copper preferred to deposit on the formed copper grains and a secondary nucleation took place in the gaps among the formed nuclei. As a result, the coalescence was obtained by the formation of some “new” nuclei in the gaps. In other words, the coalescence was achieved through a “filling in” of the gaps by new formed nuclei [15]. As shown in Table 1, although the initial copper deposition on TES-SAMs modified surface was more difficult, the deposition rate became remarkably increasing on the secondary phase for its density and crystal growth. It could be seen that the copper plating was the thickest, which would be shown in SEM images discussed later.

**Figure 1.**
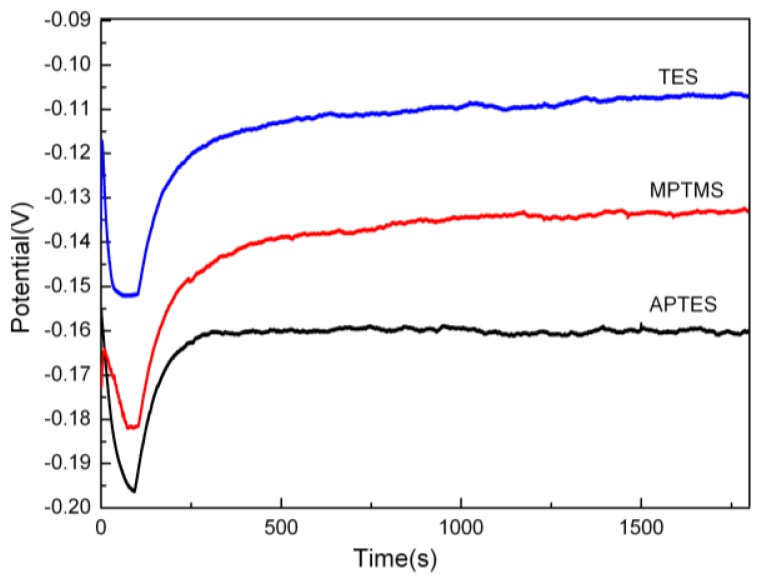
Potential curves of copper electroplating on different SAMs modified substrate.

The contact angle of copper film was measured to analyze the morphology of surface. According to the data in the Table 2, we can see that the contact angle (99.3°) and surface energy (0.176 J/m^2^) of copper film on TES-SAMs modified substrate is very approximate to the value of blank copper (pure copper plate). In general, for isotropic material, the performance of the material can be characterized by the surface contact angle Therefore, from the data of Table 2, it was inferred that the physical and electrical conductivity of deposited copper layer on TES-SAMs modified substrate was similar to that of pure copper. To further confirm the assumption, the galvanostatic method was adopted to conduct copper electroplating with the help of electrochemical workstation to depict the difference of resistance for the copper films on different SAMs modified substrate, as shown in Figure 1. The maximum potential of copper plating on TES-SAMs modified substrate was 0.152 V, while the potential on MPTMS-SAMs and APTES-SAMs modified substrate was 0.18 and 0.197 V, respectively. In general, high potential represents high resistance when the current density is constant. Therefore, the copper plating on TES-SAMs modified substrate has the minimum resistance and the best conductivity compared with those on the other two SAMs modified substrates.

### 2.2. Surface Morphology-SEM Studies for Copper Films

In a past study, the researchers found that the copper growth on SAMs modified ABS resin was easier than that on the blank (non-SAMs modified) substrate [16]. The increasing copper nucleation density and the decreasing inter-grain distance on the SAMs-modified substrate before coalescence indicated that copper atoms had a lower mobility on the SAMs-modified ABS resin compared with that on the blank substrate, and copper atoms would be immobilized by the functional groups of SAMs [17,18]. The uniform monolayers result in an equal maximum probability for copper atoms nucleation [19], which leads to forming the smooth and continuous copper film.

**Figure 2.**
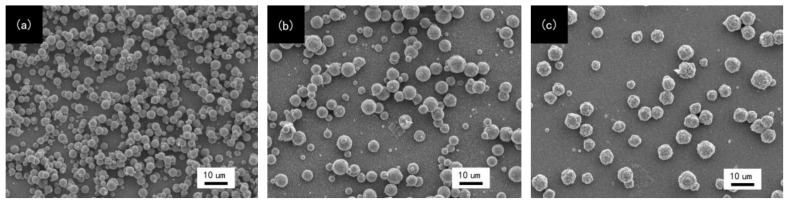
SEM images of copper film deposited on different SAMs modified ABS. (**a**) TES-SAMs modified; (**b**) MPTMS-SAMs modified; (**c**) APTES-SAMs modified.

Figure 2 shows the morphologies of the copper film deposited on different SAMs modified ABS. The property of deposited copper layer is evaluated by the comparison of copper particle coverage, which is correlated to the nucleation densities of the terminate group on different SAMs modified substrate surface. Only isolated copper particles were observed on the APTES-SAMs modified substrate, due to weaker bonding energy of terminal –NH_2_ group in the APTES-SAMs with copper. The copper nucleation densities on the MPTMS-SAMs modified substrate increased and inter-island distances decreased compared with that on APTES-SAMs modified substrate. However, the copper particle coverage was still very limited because of fewer –SH coordination sites in the MPTMS-SAMs. In contrast, it could be seen that the enhancement of copper deposition on TES-SAMs modified surface was dramatic and the size of islands was smaller. A continuous copper film formed and the film was smooth and uniform, owing to the existence of more coordination sites (ex, –SNa, –SH and triazine ring) provided by TES-SAMs. It would generate the increasing of copper nucleation tendency and improve the bonding force between copper particles. From the above analysis, it can be concluded that TES-SAMs on the substrate has the best property for enhancing the copper deposition, which makes the conductivity of the material fine.

**Figure 3.**
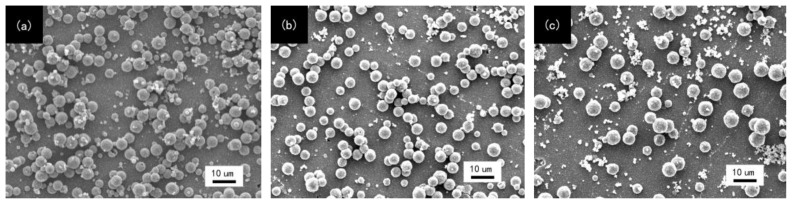
SEM images of copper film after saline solution immersion. (**a**) TES-SAMs modified; (**b**) MPTMS-SAMs modified; (**c**) APTES-SAMs modified.

To evaluate the property of copper film deposited on different SAMs modified substrate, the test of saline solution immersion was carried out. Figure 3 reveals that the density of copper islands on the substrate decreases after saline solution immersion and the copper film is even entirely corroded by saline solution. However, the copper film on TES-SAMs modified substrate still has a conspicuous advantage compared with the samples modified by MPTMS-SAMs and APTES-SAMs. In this work, the selectivity discrepancy of these different terminal group SAMs is determined by the existence of proton hydrogen in these terminal groups. In past studies, it was found that the protonation of the absorbed ligand was the rate-controlling step in copper deposition [20]. Compared with the –NH_2_ group, the proton hydrogen in –SNa and –SH groups could accelerate the protonation of the adsorbed ligand. Moreover, the –SNa group was a stronger proton-donating group than the –SH group. The result suggested that the selectivity of copper deposition derived from the ability of proton-donating for three kinds of terminal group.

### 2.3. Adhesion Property of Copper Film

**Figure 4.**
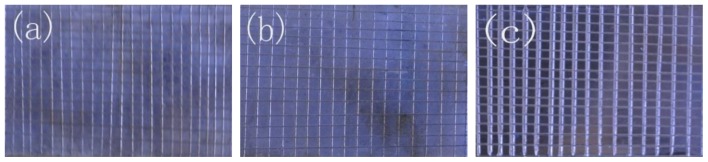
Adhesion property of copper film by cross-scratched method. (**a**) TES-SAMs modified; (**b**) MPTMS-SAMs modified; (**c**) APTES-SAMs modified.

The adhesion strength between ABS substrate and electroless copper film was a very important parameter for electroless copper deposition. The cross cut test is one of the methods to evaluate the adhesion property. Figure 4 shows that the adhesion property of the all samples modified by TES-SAMs, MPTMS-SAMs and APTES-SAMs is excellent, and almost no copper plating is stripped off from the substrate, due to the existence of the coordinate bond between copper film and the terminal group of different SAMs. Our previous study indicated that the adhesion property of copper layer would be very poor after the saline solution immersion and the copper plating layer, which was directly deposited on the blank substrate, could be completely stripped off by a tape [9]. When the samples with copper plating film are put into the environment of saline solution, water molecules or chloride ions may pass through the copper plating, initiate the corrosion of copper layer and eventually lead to the delamination of the coating. However, if there is a covalent bond or coordination bond between the copper plating and ABS substrate, it is expected that water molecules or chloride ions can not easily accumulate at the interface and delaminate the copper plating [21]. To verify the existence of covalent bond linkages or coordination bond between copper plating and SAMs, the saline solution immersion test of the samples with cross-scratch was further conducted.

**Figure 5.**
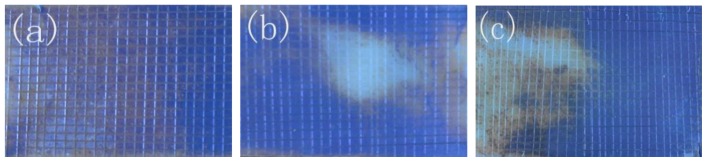
Adhesion property of copper film after saline solution immersion. (**a**) TES-SAMs modified; (**b**) MPTMS-SAMs modified; (**c**) APTES-SAMs modified.

Figure 5 shows the surface morphologies of copper film on different SAMs modified substrate after saline solution immersion. It could be observed that almost half of copper layer on APTES-SAMs modified substrate was entirely corroded and the partial substrate modified by MPTMS-SAMs was revealed. The copper plating on TES-SAMs modified substrate was still integrated and uniform after it was immersed into saline solution for 14 days. However, those unbroken copper layers deposited on the three SAMs modified substrates still possessed an outstanding adhesion property, because almost none of the rest copper plating was stripped off from the substrate. The results demonstrated the existence of covalent bond linkages or coordination bond between copper plating and SAMs. Due to the fact that the interaction between copper atom and S, N atoms of SAMs was stronger than that between copper atom and O atom on the ABS substrates, the copper plating on TES-SAMs modified substrate can not be broken in saline solution. The revealed area of the ABS substrate modified by MPTMS-SAMs and APTES-SAMs was induced by the lower coverage of copper nuclei.

### 2.4. XRD Analysis of Copper Film

**Figure 6.**
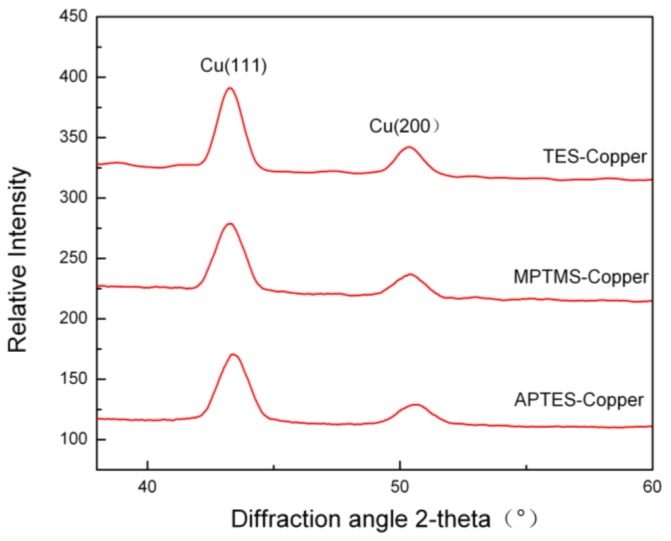
XRD patterns of copper film on different SAMs modified ABS substrate.

XRD analysis was employed to determine the microstructure of electroless copper plating. Figure 6 shows X-ray diffraction patterns of copper film on different SAMs modified substrate. The electroless copper plating exhibits a strong Cu(111) orientation with a minor contribution from the Cu(200) orientation. It was suggested that the preferential growth orientations of electroless copper film on SAM-modified ABS substrate were Cu(111). The intensity ratio *I* (111)/*I* (200) was used to evaluate the electromigration resistance life time of electroless copper films, since the electromigrtion resistance lifetime of Cu(111) is roughly longer than that of Cu(200) [22]. The intensity ratio of *I* (111)/*I* (200) of copper on TES-SAMs modified ABS (3.96) was higher than that on MPTMS (3.64) and APTES-SAMs (3.07) modified substrates. Therefore, the copper films deposited on TES-modified ABS substates should have a better capability of deterring the device failures caused by eletromigration, which means that the copper film deposited on TES-SAMs modified ABS substrates is better than those deposited on MPTMS-SAMs and APTES-SAMs modified ABS resins in electromigrtion resistance.

## 3. Experimental Section

### 3.1. Materials

**Figure 7.**
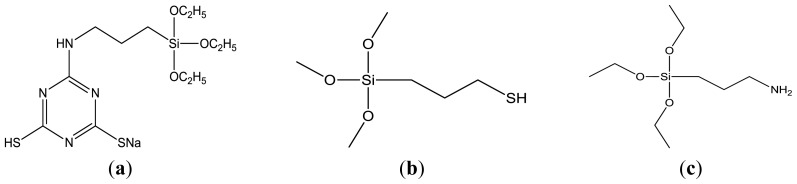
Structural formulas of TES, MPTMS and APTES. (**a**) TES; (**b**) MPTMS; (**c**) APTES.

The ABS (LG, 121H) substrates with a dimension of 50 mm × 20 mm × 1.0 mm were prepared by cutting a large plate into pieces. All test plates were degreased by ultrasonic washing in absolute ethanol for 15 min, and blow-dried in air. TES was synthesized by the reaction between 6-(3-triethoxysilylpropyl)amino-1,3,5-triazine-2,4-dichloride and NaSH according to the method in the previous study [23], MPTMS (97%) and APTES (98%) were purchased from Aladdin reagent (Shanghai, China). The structural formulas of TES, MPTMS and APTES are shown in Figure 7. Palladium chloride (BODI, Tianjin, China) and stannous chloride (BODI, Tianjin, China) crystals were stored in a vacuum dessicator prior to use. And all of the chemicals were employed as analytical reagent (AR) without further purification. Distilled water and ethanol were used as solvents.

### 3.2. Preparation of Different Self-Assembled Monolayers on ABS Surface

Firstly, the ABS resin surface was pretreated for 1 min by corona discharge using CTP-2000K (Suman Electronics, Nanjing, China). After the pretreatment, hydroxyl (–OH) or carboxyl (–COOH) was formed on the resin surface which was beneficial to react with silanol group (–Si–OH) of the hydrolyzed MPTMS, APTES or TES. Then the pretreated resin was immersed in hydrolyzed MPTMS, APTES and TES solution for 30 min at room temperature, and cured for 30 min at 90 °C in Electrothermal Constant Temp Oven (DHG-9041A; Jinghong, Shanghai, China). After that, the surface of sample was rinsed by distilled water and absolute ethanol.

### 3.3. Electroless Copper Plating

Prior to the plating step, the ABS substrates modified by different SAMs were immersed into the sensitization and activation solution composed of 0.1 g/L PdCl_2_ and 10 g/L SnCl_2_·2H_2_O for 3 min. PdCl_2_ (60%) and SnCl_2_·2H_2_O (98%) were purchased from Aladdin reagent (Shanghai, China). In order to remove the Sn^2+^ around the Pd^0^ in the colloids solution, the activated ABS resin was subsequently accelerated in an aqueous solution containing 3.7% hydrochloric acid for 30 s, and rinsed with distilled water. After that, the electroless copper plating process was conducted by immersing the surface-activated ABS resin into electroless copper plating solution for 10 min, and the composition of electroless copper plating solution was CuSO_4_·5H_2_O (16.0 g/L, BODI, Tianjin, China) as copper ion source, NaKC_4_H_4_O_6_·H_2_O (25.0 g/L, BODI, Tianjin, China) as complex agent, HCHO (25.0 mL/L, XILONG CHEMICAL, Guangdong, China, AR) as reducing agent, NH_4_Cl (2.0 g/L, BODI, Tianjin, China) as stabilizer. The pH of plating bath was adjusted to 13.0 using sodium hydroxide solution, and the bath temperature was maintained at 55 °C [9].

### 3.4. Measurement and Characterization

#### 3.4.1. Deposition Rate and Contact Angle

The deposition rate was calculated by the change in weight of ABS resin before and after the metalization process, according to the following equation.

(1) $$v=\frac{(m_{2}-m_{1})\times 10^{4}}{s\times \rho \times t}$$

where *m*_1_ and *m*_2_ represent the weight of blank and copper film plated ABS resin, respectively. *ρ* represents the density of Copper (8.96 g/cm^3^), *s* is the area of plating and *t* represents the time of electroless copper plating.

The sessile drop technique, a method often used for the characterization of solid surface energies, was applied to measure the contact angle by dropping 1 μL distilled water on the sample surface at room temperature. The water droplet formed an equilibrium dome shape on the sample surface and was observed by optical microscope with 60 times magnification. For each sample, five points were randomly chosen for measurement.

#### 3.4.2. Adhesion Property Test

The adhesion property of electroless copper plating with ABS resin was evaluated by cross cut test. The substrates covered by copper films were cross-scratched (1 mm^2^) by a single side blade, and the polyimide tape (adhesive force: 0.12 N/25 mm) was pressed on the cross-scratched area for one hour and then peeled off. Plating loss of the surface appearance was visually examined to evaluate the adhesion property. Saline solution with the concentration of 3.5% sodium chloride was used in the corrosion test. The samples were immersed into the solution for 14 days respectively. Then, the samples were rinsed and the stripping of copper layer is calculated to further evaluate the adhesion property.

#### 3.4.3. Scanning Electron Microscopy

The surface morphology of the samples was examined by scanning electron microscopy (SEM, JEOL, Beijing, China) at accelerating voltage of 25 kV.

#### 3.4.4. X-ray Diffraction

The crystal structures of electroless copper layer were measured by X-ray diffraction (XRD, D/MAX-RA, JEOL, Beijing, China) equipped with graphite monochrome Cu Kα radiation (40 kV × 100 mA, 4°/min).

## 4. Conclusions

The nucleation and growth of electroless copper plating on different terminal group modified ABS substrate were investigated. The results indicated that it was faster to form copper nuclei on the TES-SAMs modified ABS substrate compared with those on the MPTMS-SAMs and APTES-SAMs modified substrates. SEM indicated that the copper film on TES-SAMs modified substrate was more uniform and the density of copper nuclei was much higher, while the coverage of copper nuclei MPTMS-SAMs and APTES-SAMs modified substrate was very limited and the copper particle size was larger. The study for adhesion property demonstrated that all the SAMs enhanced the interfacial interaction between copper plating and substrate. The XRD analysis showed that the copper films on SAMs modified substrate had a structure with Cu(111) major orientation, and the copper film deposited on TES-SAMs modified ABS substrate is better than those deposited on MPTMS-SAMs and APTES-SAMs modified ABS resins in electromigrtion resistance.

## Figures and Tables

**Table 1.** Copper deposition rate and contact angle on different self-assembled monolayers (SAMs) covered acrylonitrile-butadiene-styrene (ABS) resin.

SAMs type	Deposition rate (nm/min)	Contact angle (°)	Surface energy (J/m^2^)
TES-SAMs	144 ± 10	71.9 ± 2	0.430 ± 0.01
MPTMS-SAMs	95 ± 10	78.9 ± 3	0.350 ± 0.02
APTES-SAMs	72 ± 10	87.8 ± 2	0.269 ± 0.01

**Table 2.** Contact angle and surface energy of copper films on different SAMs.

Copper plating	Contact angle (°)	Surface energy (J/m^2^)
blank copper	94.3 ± 3	0.218 ± 0.02
TES-copper	99.3 ± 3	0.176 ± 0.02
MPTMS-copper	114 ± 3	0.092 ± 0.02
APTES-copper	122 ± 4	0.041 ± 0.01
